# mRNA-based approach to monitor recombinant gamma-interferon restoration of LPS-induced endotoxin tolerance

**DOI:** 10.1186/cc10513

**Published:** 2011-10-25

**Authors:** Fanny Turrel-Davin, Fabienne Venet, Cécile Monnin, Véronique Barbalat, Elisabeth Cerrato, Alexandre Pachot, Alain Lepape, Christine Alberti-Segui, Guillaume Monneret

**Affiliations:** 1 BioMérieux, Joint Unit Hospices Civils de Lyon, Hôpital Edouard Herriot, 69003 Lyon, France; 2 Cellular Immunology Laboratory, Hospices Civils de Lyon, Hôpital Edouard Herriot, 69003 Lyon, France; 3 BioMérieux sa, F-69280 Marcy l'Etoile, France; 4 Intensive Care Unit, Hospices Civils de Lyon, Centre Hospitalier Lyon-Sud, Chemin du Grand Revoyet, 69495 Pierre-Bénite, France

## Abstract

**Introduction:**

It is now well accepted that sepsis is associated with the development of a pronounced immunosuppressive state, characterized by severe immune alterations (e.g. reduced proliferative capacity, endotoxin tolerance, apoptosis) participating in increased mortality and susceptibility to nosocomial infections. Efforts are currently aimed at restoring a functional immune response in septic patients. Successful therapy depends on the identification of appropriate immunostimulatory drugs and on the development of suitable biomarkers that could be used to stratify patients and to follow response to treatment.

**Methods:**

In this study, we evaluated the *ex vivo *effect of recombinant interferon gamma (rIFN-γ) in restoring monocyte functionality (endotoxin-induced Tumor Necrosis Factor-α production) in a two-hit model of endotoxin tolerance (ET) with peripheral blood mononuclear cells from healthy volunteers and in whole blood of septic shock patients. Importantly, we used quantitative-reverse transcription polymerase-chain reaction to monitor the effect of rIFN-γ on the expression of seven genes known to participate in ET (*TNF-α*, *IL-10*, *HLA-DRA*, *CIITA*, *IRAK-M*, *ABIN-3 *and *LY64)*.

**Results:**

Expression analysis of those genes confirmed the presence of an immunosuppression state and the ex vivo restoration of immune functions by rIFN-γ. We show for the first time that rIFN-γ is able to bypass, at the mRNA level, the effect of negative regulators of the LPS signalling pathway such as *IRAK-M, ABIN-3 *and *LY*6*4*.

**Conclusions:**

Overall, mRNA expressions of a panel of genes could represent promising candidates for the *ex vivo *evaluation of rIFN-γ effect on monocyte functionality. This ex vivo translational research study demonstrates the potential of a mRNA-based approach to successfully monitor drug efficacy.

## Introduction

Despite advances in supportive care and a number of clinical trials, sepsis remains the leading cause of death in non-coronary ICUs [1].

With a better understanding of the pathophysiology of sepsis, it is now evident that the early pro-inflammatory phase of the disease is immediately followed by an anti-inflammatory response that rapidly results in an immunosuppressive state. Immunosuppression is believed to be responsible for the increased risk of nosocomial infections and mortality [1-3] and represents an innovative target for future clinical trials. Current challenges consist of finding appropriate immunostimulant drugs, identifying patients that would benefit from immunomodulatory therapies (tailored immunotherapy) and monitoring successful response to treatment. As suggested by Carlet *et al*. [4], the development of biological models representative of the immunosuppressive state of the disease and the use of biomarkers may facilitate testing of immunostimulant drugs and the monitoring of response to treatment.

Among other alterations, sepsis-induced immunosuppression is characterized by dramatic monocyte/macrophage dysfunctions [1-3,5]. The intensity of such dysfunctions has been correlated with an increased risk of death and nosocomial infections in septic patients. Interestingly, these alterations have been partly reproduced in an *ex vivo *model of endotoxin tolerance (ET). Indeed, e*x vivo *prior exposure of innate immune cells to minute amounts of endotoxin causes a temporary insensitivity and renders cells refractory to subsequent lipopolysaccharide (LPS) challenge [6,7]. As observed in patients, this phenomenon is associated with monocyte/macrophage functional alterations, including a reduction in the production of pro-inflammatory cytokines associated with an increased expression of anti-inflammatory cytokines [6-10] and a decrease in antigen presenting capacity partly due to a reduced HLA-DR expression [11-15]. Moreover, LPS unresponsiveness is associated with the upregulation of numerous mechanisms that negatively regulate toll-like receptor (TLR)-associated signaling pathways [6,7].

In this study we evaluated in an *ex vivo *experimental model of ET the potential of an immunostimulating therapy (recombinant Interferon (IFN)-γ) that has been proposed as a potential innovative treatment for sepsis [11-13]. Importantly, and as a proof of concept, we monitored response to treatment through the expression level of genes that have been shown in the literature to be involved in ET (*TNF-α, IL-10, HLA-DRA, CIITA, LY64, IRAK-M *and *ABIN-3*). Using gene expression analysis we confirmed the inflammatory properties of rIFN-γ. More importantly, restoration of ET-induced monocyte dysfunction by rIFN-γ was found in clinical samples from septic shock patients.

## Materials and methods

### Preparation of PBMCs and experimental settings of ET model

**Figure 1:**
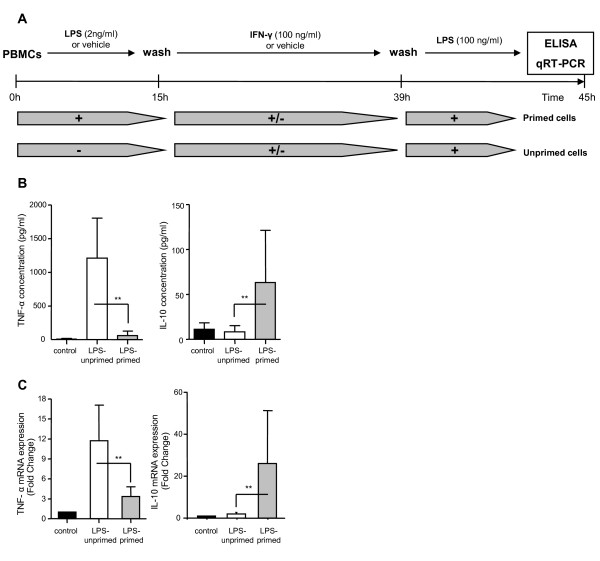
**TNF-α and IL-10 protein and mRNA expressions in the *ex vivo *model of endotoxin tolerance**. **(a) **Schematic representation of the endotoxin tolerance (ET) model used in the present study. **(b) **TNF-α and IL-10 protein concentrations and **(c) **mRNA expressions were assessed, respectively, by ELISA in culture supernatant or by quantitative RT-PCR in cell pellet obtained in peripheral blood mononuclear cells (PBMCs) after ET induction. Mean (± standard deviation) data from 10 independent experiments in healthy volunteers are given. Gene expression results are presented as a normalized ratio vs data obtained in unstimulated cells (fold changes vs control). Black columns represent controls (cells without any lipopolysaccharide (LPS) stimulation), white columns indicate LPS-unprimed cells (only stimulated once with 100 ng/ml LPS) and grey columns show LPS-primed cells (two LPS stimulations: 2 ng/ml followed by 100 ng/ml). The Wilcoxon signed rank test was used to test for statistical significance (** *P *< 0.005 vs LPS-unprimed cells). IFN, interferon.

As shown in Figure 1a, human peripheral blood mononuclear cells (PBMCs) from healthy volunteers were isolated from citrated venous blood by Ficoll-Paque density gradient centrifugation (Amersham Biosciences, Björkgatan, Sweden) and washed with PBS while the remaining red blood cells were lysed. Cells were cultured in 24-well plates at 2 × 10^6 ^cells/ml in X-Vivo 20 Medium (Lonza, Verviers, Belgium). Blood from healthy volunteers was obtained from EFS (Etablissement Francais du Sang). To induce the LPS-primed state, PBMCs were cultured in the presence or absence (control group) of 2 ng/ml LPS mix from *Escherichia coli *O55:B5, O127:B8 and O111:B4 (Sigma-Aldrich, Deisenhofen, Germany) and incubated overnight at 37°C and 5% CO2 (15 hours). Following a washing step, PBMCs were incubated for an additional 24 hours in the presence or absence (control group stimulated or not with LPS) of rIFN-γ1b (Imukin, Boehringer, Ingelheim, Austria). Finally, cells were stimulated a second time by adding 100 ng/ml of LPS for another six hours. TNF-α and IL-10 protein concentrations in those supernatants were assessed as well as mRNA expressions in cell pellets. For each condition, supernatants and cell pellets were recovered and stored at -80°C for TNF-α and IL-10 measurements by ELISA and at -20°C for RNA extractions and quantifications by quantitative-reverse transcription polymerase chain reaction (qRT-PCR), respectively.

### Functional testing by ELISA

Detection of TNF-α and IL-10 concentrations in PBMCs culture supernatants was done by using commercially available ELISA kits from R&D System (Minneapolis, MN, USA).

### RNA extraction and cDNA synthesis by reverse transcription

Total RNA was extracted from PBMCs using RNeasy Plus Mini kits (Qiagen, Hilden, Germany) or from whole blood using QIAamp RNA Blood Mini Kit (Qiagen, Hilden, Germany). For each RNA extraction, the residual genomic DNA was digested using the gDNA Eliminator spin column (Qiagen, Hilden, Germany). RNA was diluted in 30 μl of elution buffer. RNA quantity was determined for each sample using a Qubit (Invitrogen, Carlsbad, CA, USA) according to the manufacturer's instructions. Then, cDNA was synthesized from 100 ng of total RNA using SuperScript^® ^VILO™ system (Invitrogen, Carlsbad, CA, USA) according to manufacturer's instructions.

### Quantitative PCR analysis

mRNA expression level was quantified using qRT-PCR. Briefly, PCR reactions were performed in a LightCycler^® ^480 instrument using the associated SYBR Green I Master Mix according to the manufacturer's instructions (Roche Molecular Biochemicals, Indianapolis, IN, USA). For amplification, the reaction volume was 20 μl, and the cycling conditions were as follows: an initial denaturation step at 95°C for five minutes (one cycle), followed by 45 cycles of a touch-down PCR protocol (20 seconds at 95°C, 15 seconds annealing at 68 to 58°C and 15 seconds extension at 72°C), a melting curve at 95°C for one second, 60°C for 10 seconds and 95°C for five minutes, and to finish a cooling at 40°C for 30 seconds. mRNA expression levels of the housekeeping gene *Peptidylpropyl isomerase B *(*PPIB*), encoding for cyclophilin B, and *TNF-α *were investigated using specific cDNA standards and ready-to-use primer mixes obtained from Search-LC (Search-LC, Heidelberg, Germany). The efficiency of *PPIB *mRNA levels as reference for target mRNA quantification has been previously demonstrated in human peripheral blood [16]. The PCR amplicons of the genes of interest (*IL-10, HLA-DRA, CIITA, LY64, IRAK-M *and *ABIN-3*) were obtained with the primer combinations presented in Table 1. Serial dilutions of the cDNA were prepared in quadruplicate to generate standard curves. Relative standard curves, describing the PCR efficiency of the genes panel and *PPIB*, were created and used to perform efficiency-corrected quantification with the LightCycler Software version 1.5. An internal calibrator was used to compare each cDNA amplification. The results are expressed as fold change normalized ratio of *TNF-α, IL-10, HLA-DRA, CIITA, LY64, IRAK-M *and *ABIN-3 *mRNA relative to *PPIB *mRNA. qRT-PCR results were included in the analysis only when *PPIB *and target mRNA values were comprised within the standard curve.

**Table 1:** Quantitative PCR performance, parameters and primers

Gene symbol	GenBank	**Primer sequences **^**a**^
*IL-10*	NM_000572.2	5'-AATAAGGTTTCTCAAGGGGCT-3'
		5'-AGAACCAAGACCCAGACATCAA-3'
*HLA-DRA*	NM_019111.4	5'-GCCAACCTGGAAATCATGACA-3'
		5'-AGGGCTGTTCGTGAGCACA-3'
*CIITA*	NM_000246.3	5'-GCTGGGATTCCTACACAATGC-3'
		5'-CGGGTTCTGAGTAGAGCTCAATCT-3'
*IRAK-M*	NM_007199.2	5'-TTTGAATGCAGCCAGTCTGA-3'
	NM_001142523.1	5'-GCATTGCTTATGGAGCCAAT-3'
*ABIN-3*	NM_024873.4	5'-GAATTCCCAGATAAAAGCTTGT-3'
	NM_001128843.1	5'-GACAGTCTGGTGGGTGCTC-3'
*LY64*	NM_005582.2	5'-GCATTGAGAAAGAAGCCAACAA-3'
		5'-GAAAAGTGTCTTCATGTATCC-3'

^a ^Top sequence is forward primer, bottom is reverse

### Septic shock patients and whole blood model

The study group consisted of eight consecutive septic shock patients according to the diagnostic criteria of the American College of Chest Physicians/Society of Critical Care Medicine [17]. Patients were admitted to the two participating ICUs (one medical, one surgical) of the Lyon-Sud University Hospital (Hospices Civils de Lyon, France). Septic shock was defined by an identifiable site of infection, evidence of a systemic inflammatory response manifested by at least two of the following criteria: a) temperature more than 38°C or less than 36°C; b) heart rate above 90 beats per minute; c) respiratory rate above 20 breaths per minute; d) white blood cell count above 12,000 cells/mm^3 ^or less than 4,000 cells/mm^3 ^and hypotension persisting despite fluid resuscitation and requiring vasopressor therapy. The onset of septic shock was defined by the beginning of vasopressive therapy. The only exclusion criteria were patients younger than 18 years old and the absence of circulating leukocytes. Patients were treated according to the standardized recommendations of our ICUs. Severity at the onset of shock was assessed by the Simplified Acute Physiology Score II (SAPS II, range: 0 to 194) [18]. Development of organ dysfunctions was assessed by the Sequential Organ Failure Assessment score (SOFA, range: 0 to 24) measured after 24 hours of ICU stay [19].

EDTA whole blood was collected from eight patients within three days after the onset of shock and from eight healthy volunteers. After centrifugation and removal of plasma, 3 ml of blood was diluted 1:1 with 3 ml of RPMI 1640 medium (Eurobio, Courtaboeuf, France) and then cultured in presence or absence (control group) of 100 ng/ml LPS +/- 100 ng/ml rIFN-γ1b (Imukin, Boehringer, Ingelheim, Austria) overnight at 37°C and 5% CO2 (15 hours). For each condition, supernatants and cell pellets were recovered and stored at -80°C for TNF-α measurement by ELISA and at -20°C for RNA extraction and quantification by qRT-PCR, respectively, as described previously in this manuscript.

### Flow cytometry on peripheral blood from patients

Flow cytometric (FC500, Beckman-Coulter, Hialeah, FL, USA) expressions of cell surface markers was assessed on EDTA-anticoagulated peripheral blood from patients. Monoclonal antibodies and their respective isotype controls were used according to manufacturer's recommendation: PE-labeled anti-HLA-DR (Becton Dickinson-Pharmingen, San Jose, CA, USA), FITC-labeled anti-CD14, ECD-labeled anti-CD4, PE-labeled anti-CD127, PECy5-labeled anti-CD25 (Immunotech, Marseille, France). Red blood cells were lysed using the automated TQ-Prep lysing system (Beckman-Coulter, Miami, FL, USA) in the case of Treg measurement or using FACS lysing solution (Becton Dickinson-Pharmingen, San Jose, CA, USA) for HLA-DR. Results are expressed as percentages of CD4^+^CD25^+^CD127^- ^cells out of the total CD4^+ ^lymphocytes and as percentages of cells expressing HLA-DR among total monocyte population. This work belongs to a global study on ICU-induced immune dysfunctions. It has been approved by our Institutional Review Board for ethics which waived the need for informed consent because biomarkers expression was measured on residual blood after completing routine follow-up. This study is registered at French Ministry of Research and Enseignement (#DC-2008-509). It is also recorded at the Comission Nationale de l'Informatique et des Libertés.

### Statistical analysis

Results were expressed as mean ± standard deviation. Statistical analysis was performed using the non-parametric Wilcoxon paired test for comparison between culture conditions or using the Mann Whitney U-test for comparison between septic patients and healthy volunteers. A *P *value less than 0.05 was considered significant with correction by the number of analyses performed.

## Results

### Description of the model of endotoxin tolerance (ET)

Models of ET have been characterized by a reduction of TNF-α production associated with an increase in IL-10 secretion following a secondary stimulation with LPS. These cytokines were therefore measured in healthy volunteers' PBMCs supernatant after two challenges with LPS (primed cells) in comparison with cells that were only stimulated with LPS at 100 μg/ml (unprimed cells - Figure 1a). As expected, in response to a second LPS challenge, LPS-primed cells released less TNF-α (58.5 ± 68.3 pg/mL) than unprimed cells (1208 ± 594 pg/mL) and produced more IL-10 (63.2 ± 58.2 vs 8.4 ± 6.9 pg/mL in unprimed cells) (Figure 1b). These results were confirmed at the mRNA level (Figure 1c).

Therefore, this inability to produce TNF-α combined with an increase of IL-10 production in response to a secondary endotoxin challenge in LPS-primed cells confirmed the development of an ET state in our model.

### rIFN-γ significantly improves TNF-α production in LPS-deactivated PBMCs

Recombinant IFN-γ has been proposed as a potential immunostimulating therapy in septic shock or trauma patients and preliminary clinical trials have provided encouraging results [11-13]. Therefore, we tested the effect of this drug in our model of ET.

**Figure 2:**
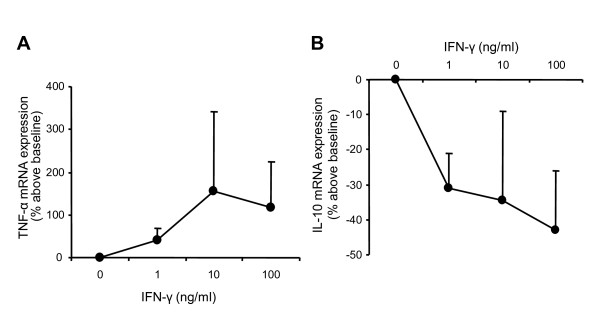
**Recombinant IFN-γ dose-response curves**. mRNA expressions of **(a) ***TNF-α *and **(b) ***IL-10 *were quantified by quantitative RT-PCR in peripheral blood mononuclear cells (PBMCs) from four healthy volunteers after lipopolysaccharide (LPS) incubation (2 ng/ml for 15 hours) and treatment with or without (baseline) rIFN-γ1b (at 1, 10 or 100 ng/ml) for 24 hours. Values are presented as means and standard deviations of percentage above baseline. IFN, interferon.

In four preliminary experiments in healthy volunteers' PBMCs incubated with a single dose of LPS (2 ng/ml), dose effect of rIFN-γ on *TNF-α *and *IL-10 *gene expression levels was investigated. As shown in Figure 2, rIFN-γ dose-dependently increased *TNF-α *and decreased *IL-10 *mRNA expression levels. On the basis of these results and in agreement with previous reports [11,20-22], cells were incubated with a drug dosage of 100 ng/ml in all further experiments.

**Figure 3:**
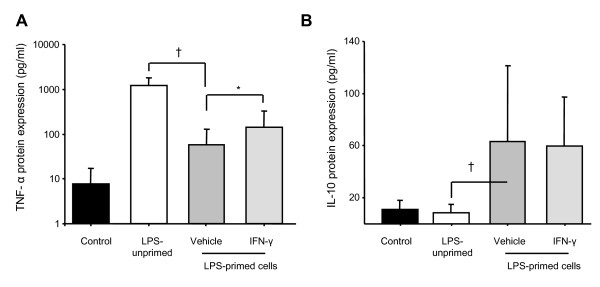
**Effect of recombinant IFN-γ on TNF-α and IL-10 protein expressions**. **(a) **TNF-α and **(b) **IL-10 protein concentrations were assessed by ELISA in culture supernatants obtained after endotoxin tolerance (ET) induction in peripheral blood mononuclear cells (PBMCs) from 10 healthy individuals. Mean (± standard deviation) data are given. Black columns represent controls (cells without any lipopolysaccharide (LPS) stimulation), white columns indicate LPS-unprimed cells (only stimulated once with 100 ng/ml LPS) and dark gray columns show LPS-primed cells (two LPS stimulations: 2 ng/ml followed by 100 ng/ml) and light grey show LPS-primed cells incubated with rIFN-γ1b (100 ng/ml). The Wilcoxon signed rank test was used to test for statistical significance († *P *< 0.005 vs LPS-unprimed cells; * *P *< 0.05 vs vehicle). IFN, interferon.

**Figure 4:**
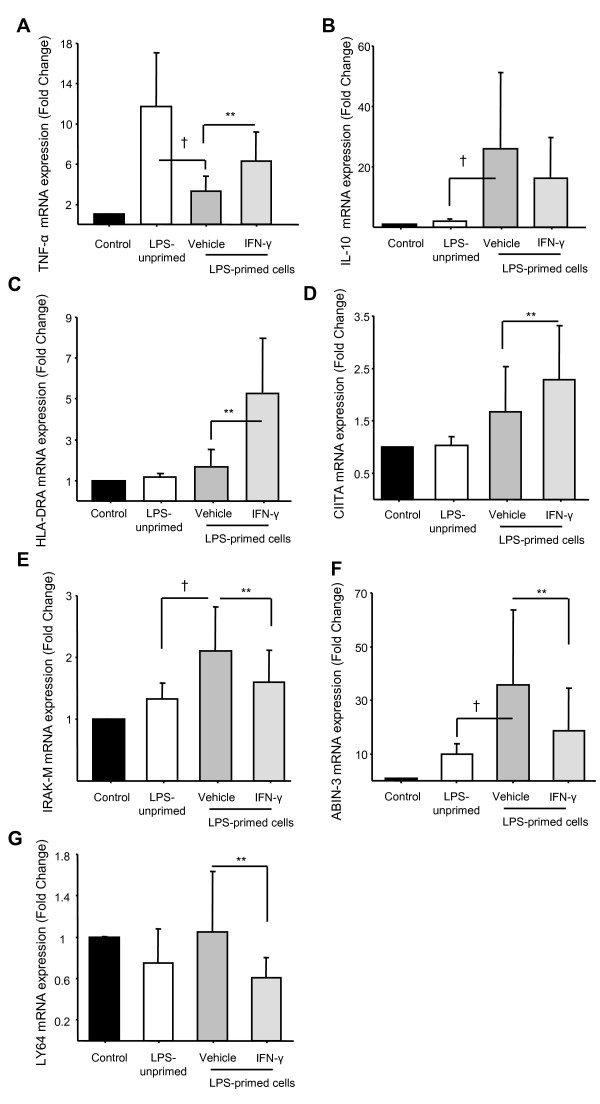
**mRNA expression analysis of seven genes after incubation with recombinant IFN-γ in the *ex vivo *model of endotoxin tolerance**. mRNA expressions of **(a) ***TNF-α*, **(b) ***IL-10*, **(c) ***HLA-DRA*, **(d) ***CIITA*, **(e) ***IRAK-M*, **(f) ***ABIN-3 *and **(g) ***LY64 *were quantified by quantitative RT-PCR in peripheral blood mononuclear cells (PBMCs) from 10 healthy volunteers after induction of endotoxin tolerance. Black columns represent controls (cells without any lipopolysaccharide (LPS) stimulation), white columns indicate LPS-unprimed cells (only stimulated once with 100 ng/ml LPS) and dark grey columns show LPS-primed cells (two LPS stimulations: 2 ng/ml followed by 100 ng/ml) and light grey show LPS-primed cells incubated with rIFN-γ1b (100 ng/ml). Gene expression results are presented as a normalized ratio vs data obtained in unstimulated cells (fold changes vs control). Data are presented as mean ± standard deviation. The Wilcoxon signed rank test was used to test for statistical significance († *P *< 0.005 vs LPS-unprimed cells; ** *P *< 0.005 vs vehicle). IFN, interferon.

We next investigated the effect of rIFN-γ in 10 successive ET experiments in PBMCs from healthy volunteers. As expected, we observed that incubation of primed cells with rIFN-γ before the second LPS challenge was associated with a significant increase of TNF-α concentration in the supernatant (Figure 3a). Interestingly, no effect was observed on IL-10 concentration (Figure 3b). These results were confirmed at the mRNA level as we observed a significant up-regulation of *TNF-α *mRNA expression following rIFN-γ challenge compared with control values (Figure 4a). As shown in Figure 4b, no variation in the mRNA expression level of *IL-10 *was observed.

In addition, we monitored by qRT-PCR the expression of five genes described in the literature to be involved in the ET refractory state or sepsis-induced immunosuppression (*HLA-DRA, CIITA, IRAK-M, ABIN-3*, and *LY64*) [7]. Our results showed that, in LPS-primed cells, rIFN-γ induced a significant up-regulation of *HLA-DRA *and *CIITA *mRNA expressions (Figures 4c and 4d) associated with a significant decrease in the expression level of *IRAK-M*, *ABIN-3 *and *LY64 *(Figures 4e to 4g).

### rIFN-γ effect on septic shock patient's whole blood

**Figure 5:**
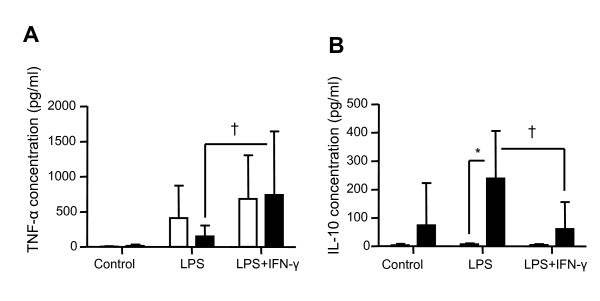
**TNF-α and IL-10 productions after incubation with recombinant IFN-γ and LPS in whole blood from septic shock patients**. **(a) **TNF-α and **(b) **IL-10 protein expressions were assessed by ELISA, in EDTA whole blood supernatants after incubation with or without lipopolysaccharide (LPS; 100 ng/ml) and rIFN-γ (100 ng/ml). Results were obtained from eight septic shock patients sampled within three days after the onset of shock (black columns) and eight healthy volunteers (open columns) stimulated or not (control group) with LPS in absence or in presence of rIFN-γ overnight. Data are presented as mean ± standard deviation. Comparison between healthy volunteers and septic shock patients was performed using the Mann-Whitney U test (* *P *< 0.05) whereas evaluation of rIFN-y effect was performed using the Wilcoxon signed rank test († *P *< 0.05). IFN, interferon.

**Figure 6:**
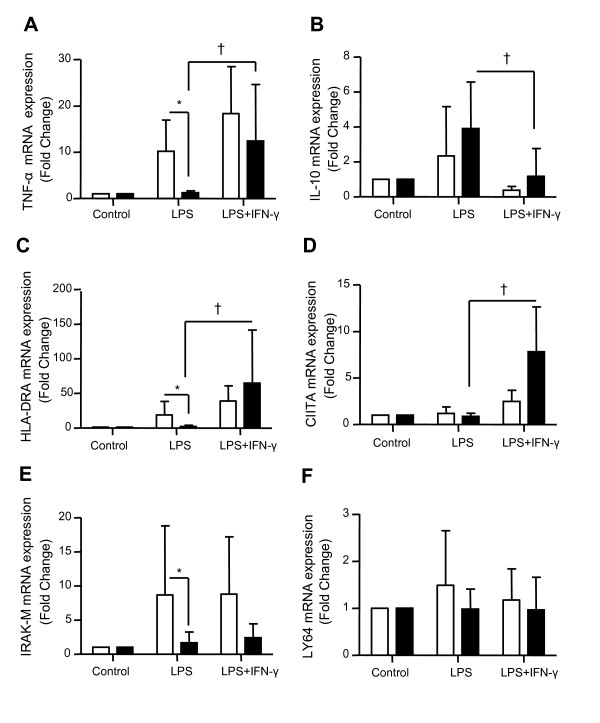
**Gene expression restoration after incubation with recombinant IFN-γ in whole blood of septic shock patients**. Gene expressions of **(a) ***TNF-α*, **(b) ***IL-10*, **(c) ***HLA-DRA*, **(d) ***CIITA*, **(e) ***IRAK-M *and **(f) ***LY64 *were quantified by quantitative RT-PCR in EDTA whole blood from eight septic shock patients sampled within three days after the onset of shock (black columns) and eight healthy volunteers (open columns), stimulated or not (control) with lipopolysaccharide (LPS; 100 ng/ml) ± rIFN-γ1b (100 ng/ml) for one night. Data are presented as mean ± standard deviation. Comparison between healthy volunteers and septic shock patients treated with LPS was performed using the Mann-Whitney U test (* *P *< 0.05) whereas evaluation of rIFN-y effect was performed using the Wilcoxon signed rank test († *P *< 0.05). IFN, interferon.

Finally, EDTA whole blood from eight septic shock patients sampled within three days after the onset of shock was incubated with rIFN-γ. Clinical data are presented in Table 2. At the time of sampling, these patients presented with a reduced percentage of HLA-DR expressing monocytes (60%/39-67), Median/Q1-Q3), a marked lymphopenia (0.6 × 10^3 ^lymphocytes/μl (0.6-1)) and an increased percentage of circulating Treg cells (11%/10-13). As expected, in response to LPS, patients produced lower amount of TNF-α and significantly more IL-10 protein than healthy controls (Figures 5a and 5b). These results were confirmed at the mRNA level (Figures 6a and 6b). Importantly, the addition of rIFN-γ significantly restored TNF-α and abrogated IL-10 productions (Figures 5a and 5b). Those variations were observed at the mRNA level in septic shock patients (Figures 6a and 6b). Moreover and as observed in our model of ET, rIFN-γ induced a massive elevation of *HLA-DRA *and *CIITA *mRNA expression levels but had no effect on *IRAK-M *and *LY64 *(Figures 6c to 6f). *ABIN-3 *mRNA expression level has a similar behavior as *IRAK-M*. However, we could not conclude on *ABIN-3 *since its mRNA values for control conditions (nonstimulated cells) were below the lowest values of the standard curve (data not shown).

**Table 2:** Demographic and clinical data for septic shock patients

Parameters	Patients (*n *= 8)
**Age at admission **(years)	75 (64-79)
**Gender **(male)	6 (75%)
**SOFA score**	11 (11-12)
**SAPS II score**	55 (48-64)
**% HLA-DR^+ ^monocytes**	60 (39-67)
**% of regulatory T cells **(among CD4^+ ^lymphocytes)	11 (10-13)
**Number of lymphocytes **(10^3^/μl)	0.6 (0.6-1)
**Sampling time **(Days after shock)	1 (1-1)
**Mortality **(survivors)	4 (50%)

Results are presented as median and interquartile range (Q1-Q3) for continuous variables or as number of cases and percentages (%) for categorical data.

SAPS II, Simplified Acute Physiology Score II measured on admission; SOFA score, Sequential Organ Failure Assessment measured during the first 24 hours of ICU stay.

## Discussion

It is now largely accepted that sepsis is characterized by the development of profound immune dysfunctions [1,2,23]. Such alterations could explain patients' inability to fight the primary bacterial infection and their decreased resistance to secondary nosocomial infections. As a consequence, sepsis-induced immune dysfunctions may contribute largely to mortality. Among mechanisms responsible for this immunosuppression, monocyte dysfunction, generically called ET, is believed to play a pivotal role [2,6,7,24]. Clinically, the state of ET is associated with functional and phenotypic alterations of circulating monocytes. It has been demonstrated that patients with the most severe monocyte dysfunctions are those who have a greater risk of developing nosocomial infections and a greater risk of dying [14,15,25]. Overall, these observations provide a rational for the development of novel therapies aimed at boosting the immune functions in septic patients.

ET can be partly mimicked *ex vivo *when cells from healthy volunteers are activated by sequential LPS challenges [24,26,27]. Here, we took advantage of this model to investigate the effects of a pro-inflammatory drug, rIFN-γ, which previously provided interesting preliminary results in small clinical studies [11-13]. We assessed *ex vivo *drug effect through the monitoring of the expression level of a panel of seven genes (i.e. *TNF-α, IL-10, HLA-DRA, CIITA, IRAK-M, ABIN-3 *and *LY64)*.

In the present experiments, we observed that rIFN-γ restored *ex vivo *the production of the pro-inflammatory cytokine TNF-α, therefore reflecting monocyte function improvement. We observed an increase in the expressions of *TNF-α*, *HLA-DRA *and *CIITA *mRNA following rIFN-γ treatment in our model of ET. Importantly, a similar increase in the expression of those genes was also observed in the whole blood of septic shock patients upon *ex vivo *rIFN-γ challenge. Our data are in agreement with previous literature [9,11]. The positive effect of rIFN-γ could be explained, in part, by the ability of IFN-γ to enhance NF-κB nuclear translocation in response to LPS [20] and to induce recruitment of transcription factors. Restoration of the accessibility to endogenous promoters, in part by facilitating TLR-induced chromatin remodeling [28], may also explain the positive effect of rIFN-γ on monocyte functionality.

Regarding the negative regulators of LPS signaling pathway, we observed, in our model of ET, an increase in the mRNA expression level of *IRAK-M *and *ABIN-3*. It is important to note that these results are in agreement with previous reports [29,30]. However, to our knowledge, we show for the first time that rIFN-γ induced *ex vivo *a restoration of the TLR signaling pathway, by decreasing both *IRAK-M *and *ABIN-3 *mRNA expression levels. Such variation in gene expression induced by rIFN-γ might add to the understanding of cell function recovery. In this model of ET, *LY64 *gene expression, which is also a TLR4 signaling pathway regulator, was decreased as well in the presence of rIFN-γ. The exact role of LY64 on TLR signaling pathways is not yet clearly established [25,31] and additional experiments are needed to further address this point.

Regarding the effect of rIFN-γ on IL-10 expression, we observed discrepant results between the ET model on healthy PBMCs and the *ex vivo *stimulation in whole blood of septic patients. Indeed, at the protein and mRNA levels, rIFN-γ did not result in a decrease of IL-10 in LPS-deactivated PBMCs. In a similar experimental model, Randow *et al*. showed an increase in the IL-10 production in refractory cells exposed to IFN-γ [22]. In contrast we found that the *IL-10 *gene was down-regulated by rIFN-γ in whole blood cells from septic shock patients. These results are in agreement with Nakos *et al*. who observed that the administration of inhaled IFN-γ in immunoparalyzed patients resulted in an increase of pro-inflammatory markers and a decrease of anti-inflammatory molecules like IL-10 [13].

Importantly, this study highlights the potential of using gene expression analysis to identify novel biomarkers that could contribute to a more personalized medicine. It is now well established that biomarkers will be useful (i) to identify patients eligible for immunomodulatory therapies, (ii) to monitor response to treatment, and (iii) to evaluate benefit/risk ratio. So far, flow cytometric measurement of HLA-DR expression on circulating monocytes has appeared as a reliable biomarker for the prediction of death and nosocomial infections in septic patients [14,15,32]. Small clinical trials have used this parameter to stratify ICU patients before administration of rIFN-γ [11,13]. However, pre-analytical and analytical issues inherent to HLA-DR measurement by flow cytometry limit its use in large multicentered clinical studies and on a routine basis [33]. Based on the results of the current study and on previous investigations showing the correlation between mRNA and protein expression levels for this parameter [32,34], *HLA-DR *and *CIITA *mRNA measurements appear as promising gene candidates for the monitoring of response to immunomodulatory therapies. This is all the more because the availability in routine labs of molecular biology platforms will enable standardized and routine use of such biomarkers.

In total, given that gene expression profiling is now recognized to offer meaningful data, it provides a new perspective in the prognosis and monitoring of septic patients and offers the foundation for possible automated tests with standardized methodologies. As an example, based on microarray data, Pachot *et al*. identified the loss of CX3CR1 as a new feature of sepsis-induced immunosuppression [25,35]. Wolk *et al*. observed that a reduction of *CD86 *mRNA expression level coupled with a low HLA-DR level is associated with an unfavorable prognosis in ICU patients with post-inflammatory immunodeficiency [36]. Recently, Hinrichs *et al*. followed the expression level of 23 mRNAs related to inflammation in patients after surgery [37]. They showed that *TNF, IL1-β, CD3D *and *PRF1 *gene expressions were significantly different in patients who developed postoperative sepsis in comparison with patients who recovered uneventfully. In addition, their results demonstrate that the combination of *TNF, IL1-β *and *CD3D *mRNA expression levels is able to predict secondary infections with an extremely good area under the curve of 0.92.

Our study has some limitations. First, as mentioned previously, seminal studies of immunostimulatory therapy in sepsis have shown that patients'stratification by their relative immunoparalysis, as evidenced by low circulating HLA-DR monocytes could improve treatment efficacy [11,13]. As this preliminary study was designed as a proof of concept to monitor response to *ex vivo *rIFN-γ through the expression level of genes, this parameter, although included in the analysis, was not a criterion for patients' inclusion. Nevertheless, at the time of sampling, mHLA-DR expression in patients was decreased in comparison with normal values (Table 2). In further studies investigating this aspect, stratification based on mHLA-DR expression should be performed and it should be investigated whether *ex vivo *rIFN-γ effect is more important in patients with low mHLA-DR expression. Second, the comparison between results in whole blood and in the model of ET shows some discrepancies. Without mentioning the difference in cell subpopulations, it is obvious that the mechanisms involved in monocyte dysfunctions partly differ between the two models. Indeed, *ex vivo *model of endotoxin tolerance uses only LPS to induce monocyte alterations whereas, in septic patients, many more parameters (IL-10, cortisol, treatments...) have been shown to impact monocyte functionality. This could explain differences between results obtained in ET model and in whole blood experiments. With that said, overall our results remain consistent between the two models.

## Conclusions

As sepsis-acquired immunosuppression appears associated with increased risk of death and of nosocomial infection, immunostimulatory approaches able to restore cell dysfunctions represent innovative and promising therapeutic strategies. In this study, we propose to follow the expression levels of a panel of genes to assess *ex vivo *monocyte-stimulating drug efficacy. The value of such biomarker panel deserves now to be evaluated in a large cohort of patients.

## Key messages

• Recombinant human IFN-γ increases *TNF-α, HLA-DRA *and *CIITA *mRNA expression levels in an *ex vivo *model of ET and in cells from septic shock patients.

• Recombinant human IFN-γ decreases *IRAK-M *and *ABIN-3 mRNA *expression levels in an *ex vivo *model of ET.

• This *ex vivo *translational research study demonstrates the potential of a mRNA-based approach to successfully monitor drug efficacy.

## Abbreviations

ELISA: enzyme-linked immunosorbent assay; ET: endotoxin tolerance; IFN: interferon; IL: interleukin; LPS: lipopolysaccharide; PBMC: peripheral blood mononuclear cells; PBS: phosphate-buffered saline; PPIB: peptidylpropyl isomerase B; qRT-PCR: quantitative reverse-transcription polymerase chain reaction; SAPS II: simplified acute physiology score II; SOFA: Sequential Organ Failure Assessment; TLR: toll-like receptor; TNF: tumor necrosis factor.

## Competing interests

The authors declare that they have no competing interests.

## Authors' contributions

FV conceived the study, participated in its design, in data analysis and drafted the manuscript. GM conceived the study, participated in its design, in data analysis, drafted the manuscript, revised the manuscript for intellectual content. CM, VB and EC participated in data acquisition and analysis. AL was involved in clinical samples and data acquisition. FTD established the PCR methodology, analysis, designed the primers, conceived the study, participated in its design, in data analysis and drafted the manuscript. AP and CAS revised the manuscript for intellectual content. All authors have read and approved the final manuscript for publication.
